# The Binaural Interaction Component in Rhesus Macaques (*Macaca mulatta*)

**DOI:** 10.1523/ENEURO.0402-21.2021

**Published:** 2021-12-15

**Authors:** John Peacock, Chase A. Mackey, Monica A. Benson, Jane A. Burton, Nathaniel T. Greene, Ramnarayan Ramachandran, Daniel J. Tollin

**Affiliations:** 1 Department of Physiology and Biophysics, University of Colorado School of Medicine, Aurora, CO 80045; 2 Neuroscience Graduate Program, Vanderbilt University, Nashville, TN 37240; 3 Department of Otolaryngology, University of Colorado School of Medicine, Aurora, CO 80045; 4 Department of Hearing and Speech Sciences, Vanderbilt University Medical Center, Nashville, TN 37212

**Keywords:** auditory brainstem response, binaural hearing, binaural interaction component, macaque

## Abstract

The binaural interaction component (BIC) is a sound-evoked electrophysiological signature of binaural processing in the auditory brainstem that has received attention as a potential biomarker for spatial hearing deficits. Yet the number of trials necessary to evoke the BIC, or its measurability, seems to vary across species: while it is easily measured in small rodents, it has proven to be highly variable and less reliably measured in humans. This has hindered its potential use as a diagnostic tool. Further measurements of the BIC across a wide range of species could help us better understand its origin and the possible reasons for the variation in its measurability. Statistical analysis on the function relating BIC DN1 amplitude and the interaural time difference has been performed in only a few small rodent species, thus it remains to be shown how the results apply to more taxonomically diverse mammals, and those with larger heads. To fill this gap, we measured BICs in rhesus macaque. We show the overall behavior of the BIC is the same as in smaller rodents, suggesting that the brainstem circuit responsible for the BIC is conserved across a wider range of mammals. We suggest that differences in measurability are likely because of differences in head size.

## Significance Statement

This article reports measurements of the binaural interaction component (BIC) of the auditory brainstem response (ABR) in rhesus macaques. Comparison with other species reveals that the behavior of the macaque BIC is similar, and, based on data available so far, statistically indistinguishable to previously measured small rodents, suggesting that the brainstem circuit that generates it is largely conserved across all rodents and primates. Differences in the measurability of the BIC are likely because of variation in head size rather than differences in the neuroanatomy.

## Introduction

The auditory brainstem response (ABR) is a non-invasive measure of auditory function recorded from scalp electrodes. ABRs comprise distinct peaks (five in humans, four in small mammals), which represent synchronized neural activity at nodes in the ascending pathway (Jewett et al., 1970). In humans, summing the monaural Waves I–IV (Waves I–III in small mammals) approximates the amplitude of the binaural-evoked ABR, indicating that these waves are generated by monaural structures. However, binaural Wave V amplitude (Wave IV in small mammals) is less than the sum of the monaural waves, suggesting that neurons producing this wave are binaural. The residual waveform after subtracting the sum of the monaural from the binaural ABRs is the binaural interaction component (BIC; Dobie and Berlin, 1979).

The BIC has received attention as an objective, noninvasive measure of binaural hearing ability. The amplitude and latency of its prominent negative peak, termed DN1, change systematically with the binaural cues to sound location: interaural differences in level (ILD) and time (ITD), and are also predictive of the perceived lateralization of a stimulus (Furst et al., 1990). Importantly, DN1 is reduced or completely absent in clinical populations that exhibit binaural hearing impairments (for review, see Laumen et al., 2016), including children with central auditory processing (Delb et al., 2003) and autism spectrum disorders (ElMoazen et al., 2020), as well as in individuals with early conductive (Gunnarson and Finitzo, 1991) and normal age-related hearing loss (Van Yper et al., 2016). Clinical detection of specific binaural hearing impairment has remained elusive as audibility is often unaffected in these populations; therefore, the BIC could serve to diagnose binaural deficits as well as delineate auditory dysfunction occurring at more peripheral brainstem levels from higher level cortical or cognitive impairment (e.g., autism and age-related hearing loss).

While the BIC holds potential as a diagnostic tool, its measurement is highly variable in humans (Sammeth et al., 2020). Understanding the specific brainstem circuits underlying the BIC could help explain its unreliable nature, improve methods for its measurement, and improve its clinical utility as a biomarker. Converging lines of evidence point to the lateral superior olive (LSO) as the circuit producing the BIC; comparative studies reveal that the BIC can be evoked in all mammalian species tested so far that possess an LSO [not all of which have a medial superior olive (MSO)] and computational studies report that LSO-like binaural excitation-inhibition processing can give rise to the BIC (Ashida et al., 2016; Benichoux et al., 2018).

The BIC has been measured in a small but growing number of species. Benichoux et al. (2018) have shown that the function relating BIC DN1 amplitude as a function of ITD is statistically the same across the tested species, suggesting conservation of the brainstem circuitry producing the BIC. However, this study only gathered data on small rodents with relatively small head sizes. Thus, it remains to be fully shown whether the results can be applied more generally across mammals, and to much larger species. In this study, we measured BICs in rhesus macaques (*Macaca mulatta*), a primate with a much larger head size than the rodents, but smaller than humans. We measured ABRs with varying ITD and examined how this compares to other species.

## Materials and Methods

### Animal preparation

Experimental procedures complied with guidelines set forth by the National Institutes of Health and approved by the Vanderbilt University Medical Center Animal Care and Use Committee. Eight normal hearing adult male rhesus macaques were used ranging in age from five to seven years and weighed 9–12 kg. The average interaural diameter did not vary much across individuals, with the mean being ∼67 mm. Monkeys were anaesthetized with ketamine (3–5 mg/kg) and dexmedetomidine (5–15 μg/kg). They were then placed in a sound treated booth (ER-247, Acoustic Systems) in a prone position. Vital signs were continuously monitored.

### Auditory Brainstem Response (ABR)

Platinum subdermal needle electrodes (F-E2-12 electrodes, Grass Technologies) were placed at the apex along the interaural axis between the ears, with a reference electrode at the nape of the neck and ground on the animal’s shoulder. Custom eartips were placed in the animals ears. The monaural and binaural ABR stimuli were designed in custom built MATLAB software (Benichoux et al., 2018). Stimuli were generated and evoked potentials recorded via an RME Fireface UCX soundcard (RME Audio). ABR potentials were collected via an WPI ISO-80 (World Precision Instruments) amplifier. Stimuli consisted of 90 dB SPL clicks presented at an average rate of 33/s. These were presented using TDT MF-1 speakers (Tucker-Davis Technologies) via the eartips. The ITD of the binaural signal was varied between ±1500 μs in steps of 500 μs. A total of 3000 repetitions of the signal were presented, interleaved, for each stimulus condition, ITD, left ear monaural, and right ear monaural. Earphones were calibrated using a Bruel & Kjær type 4182 probe microphone.

## Results

**Figure 1.**
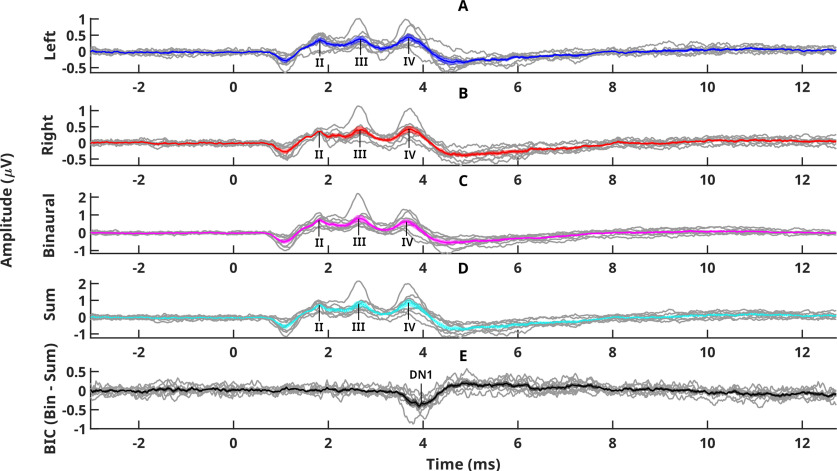
ABR waveforms for all eight monkeys. ***A***, ***B***, Averaged left and right waveforms, respectively. ***C***, Binaural signal with and ITD of 0 μs. ***D***, Sum of the two monaural responses. ***E***, BIC. The mean and SEM are shown by the colored line and the shaded area around the line. The gray lines show the data from each individual monkey. Different peaks of the waveforms are indicated.

Figure 1 shows ABR waveforms across all eight monkeys. Figure 1*A* shows averaged left ear ABRs; 1*B* the right ear, and 1*C* the binaural signal (ITD = 0 μs). Figure 1*D* shows the sum of the two monaural responses; and 1*E* shows the BIC. In all parts of the figure the shaded area shows the standard error of the mean, while the grey lines show the data from each individual monkey. ABR waveforms appeared consistent across all individuals. Averaged ABRs reveal peaks II, III and IV (wave I is difficult to see with a midline electrode montage), and the difference between the binaural and the sum of the monaural waveforms produces BIC DN1 with a latency of ∼4 ms.

**Figure 2.**
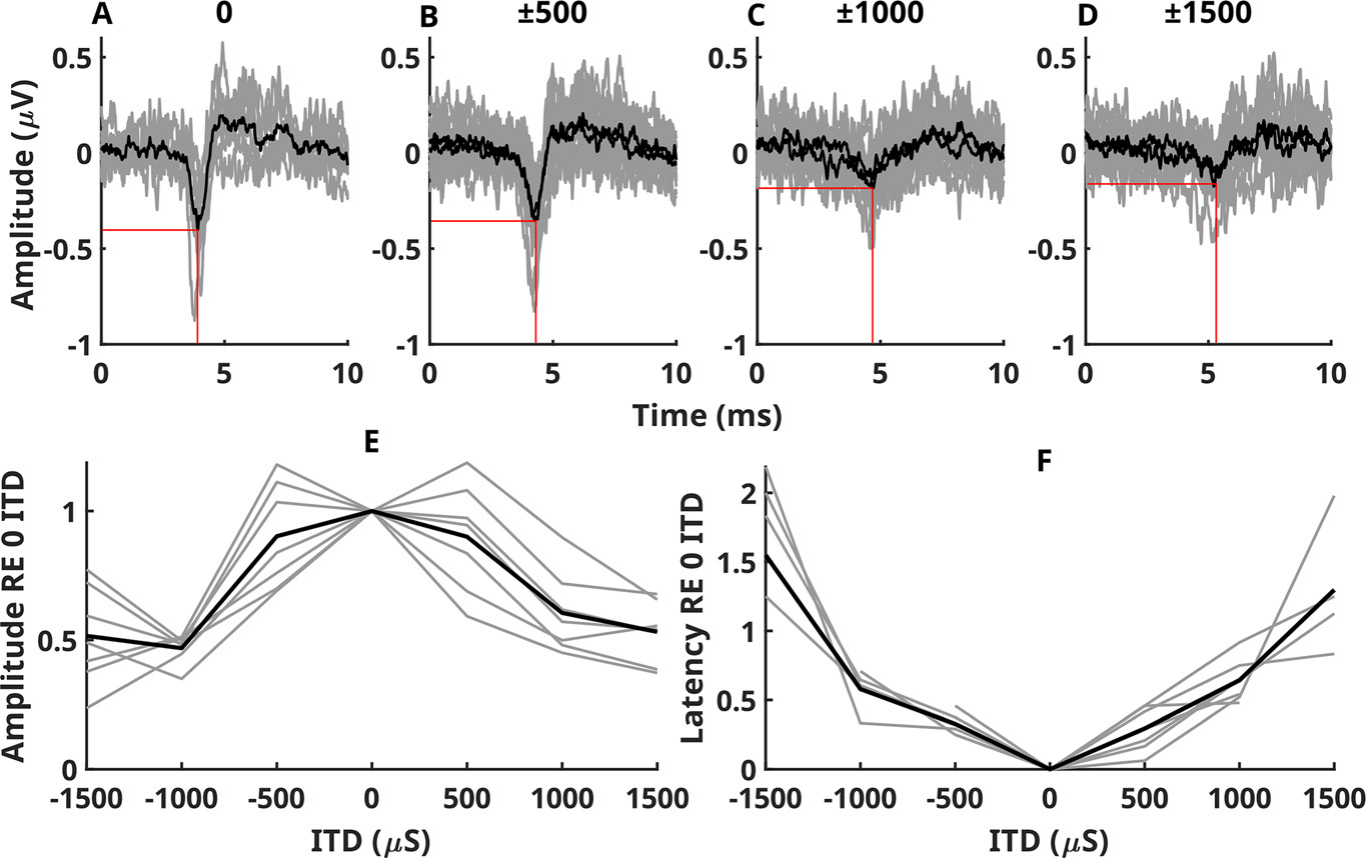
***A–D***, Raw BIC data for each measured ITD. Gray lines show measurements from individual monkeys and black lines show the mean. ***E***, ***F***, DN1 peak amplitude and latency versus ITD. ***E***, Normalized DN1 amplitudes. ***F***, DN1 latency as a function of ITD. Gray lines show data from individual monkeys, while the black line shows the mean.

Figure 2*A-D* shows the raw BIC data for each measured ITD. BICs for positive (left leading) and negative (right leading) ITDs are shown in the same panel. Grey lines show measurements from individual monkeys and black lines show the mean. BIC DN1 is visible at all ITDs, but the amplitude systematically diminishes and the latency increases with ITD. Figure 2*E/F* shows DN1 peak amplitude and latency vs ITD. Figure 2*E* shows normalised DN1 amplitudes and Figure 2*F* shows DN1 latency as a function of ITD. Grey lines show data from individual monkeys, while the black line shows the mean. On average, BIC DN1 was reduced ∼10% with ±500 μs and ∼50% by ±1000 μs ITD. Increases to ±1500 μs did not produce further reductions in DN1. Latencies of DN1 approximately followed the ITD.

**Figure 3.**
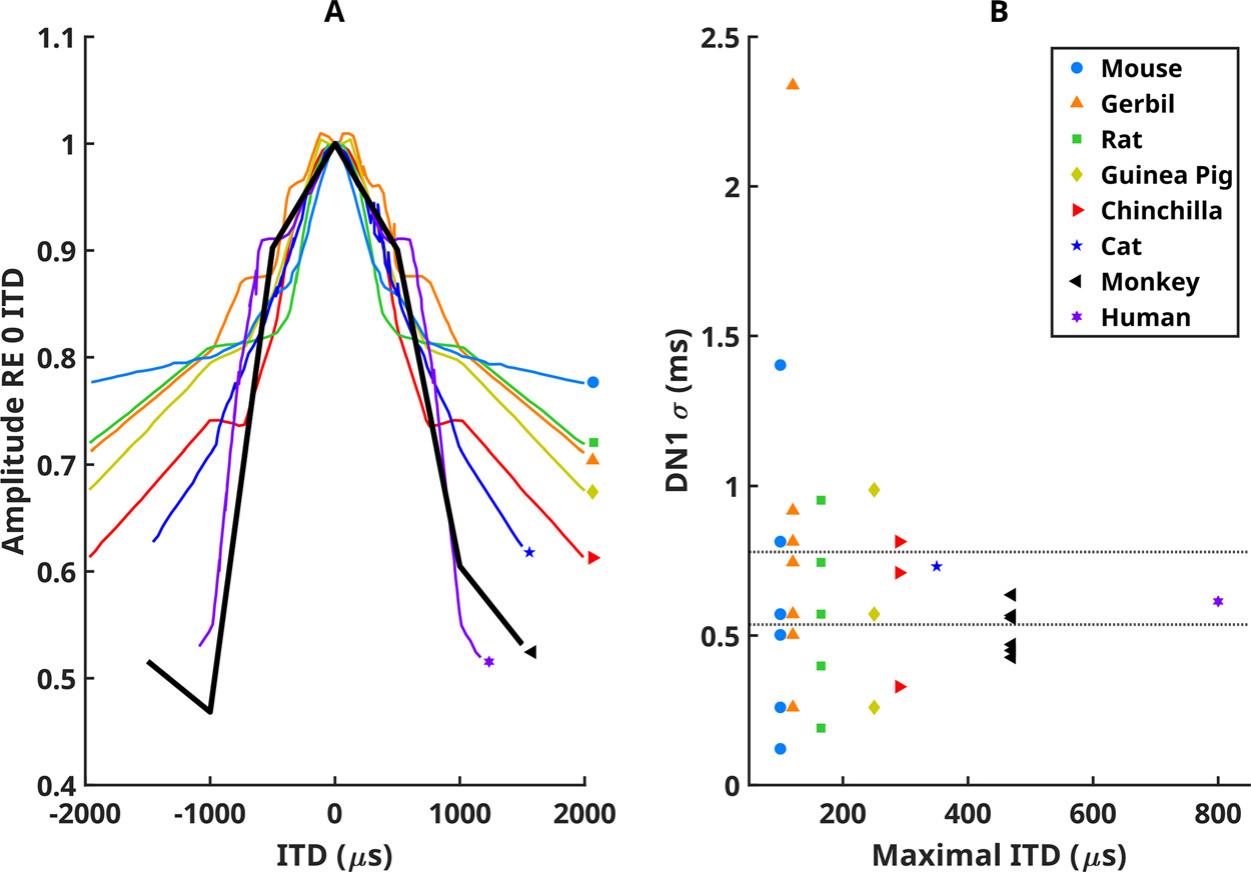
Monkey data compared to several rodent species from Benichoux et al. (2018), the domestic cat from Ungan et al. (1997), and human data from Riedel and Kollmeier (2006). Different species are indicated by different colors. ***A***, Normalized mean DN1 amplitude versus ITD for eight species. The width of a Gaussian function fitted to these data is plotted in ***B*** as a function of average maximum ITD experienced by each species.

Figure 3 compares monkey data to several rodents (Benichoux et al., 2018), the domestic cat (Ungan et al., 1997), and human data (Riedel and Kollmeier, 2006). Different species are indicated by colour. Figure 3*A* displays DN1 amplitude vs ITD for eight species. Each line shows the average of all measurements for a particular species (from Benichoux et al. (2018)). To quantify these curves, a Gaussian function was fit to the data for each individual animal. The width of the fitted curves (σ) is plotted in Figure 3*B* as a function of average maximum ITD experienced by that species.

The data from six species (excluding cat and human) were subjected to a one-way ANOVA, which revealed no significant differences in the width (σ) across species (*F*_(5,33)_ = 0.57, *p* = 0.722). The width of the curve for the cat and human were based on across-subject averages from other publications and thus were not included in the ANOVA statistical analysis. However, the 95% confidence interval calculated from the six species (Fig. 3*B*, dotted lines), bounds the cat and human data, indicating that humans and cats are not significantly different from the other species. The linear regression of the width (σ) of the DN1 versus ITD curves on the magnitude of the ITD across all eight species plotted in Figure 3*B* was not significant (*r*^2^ = 0.018, *N* = 41). This indicates that the function relating the amplitude of DN1 to ITD does not depend on the maximum ITD magnitude experienced.

## Discussion

While BIC DN1 has been examined in several mammals, many of these studies only computed DN1 for a single ITD value of 0 μs. Important clues to the brainstem mechanisms that produce the BIC can be gleaned from the function relating DN1 amplitude and latency to changes in ITD. Changes in latency can be used to test hypotheses based on existing models of brainstem ITD processing [specifically the Jeffress (1948) model], while changes in amplitude can be used to test alternative models of ITD processing based on synaptic interaction of excitation and inhibition (Ungan et al., 1997; Riedel and Kollmeier, 2006; Benichoux et al., 2018).

To date, the consensus appears to be that the DN1 latency versus ITD data are not supportive of a Jeffress-like model of ITD processing. To be consistent with Jeffress, the latency of DN1 should change at ∼1/2 the imposed ITD, but the majority of studies show DN1 latency changing at approximately the same magnitude as the ITD (see Laumen et al., 2016) . DN1 is therefore unlikely to be elicited through the brainstem circuit comprising the MSO, which has traditionally been assigned the responsibility of encoding the ITD cues.

The bulk of the current evidence supports the hypothesis that the LSO, not the MSO, is the source of the BIC. Traditionally, the LSO has been thought to be the brainstem circuit responsible for encoding the ILD cue to location (Tollin, 2003; Owrutsky et al., 2021). Recently, Benichoux et al. (2018) showed that the function relating the amplitude of BIC DN1 to ITD was statistically indistinguishable across several rodent species, including in two species that do not have a binaurally functional MSO (mice and rats). Benichoux et al. (2018) examined this data using a computational model of the LSO similar to those suggested earlier by Ungan et al. (1997) and Riedel and Kollmeier (2006), and more recently formalized by Ashida et al. (2016, 2017). A more direct study demonstrated that DN1 derived from the multiunit spiking of LSO neurons recorded *in vivo* exhibits the same ITD dependence as the simultaneously measured ABR-derived BIC, while DN1 derived from MSO neurons recorded *in vivo* does not (Tolnai and Klump, 2020). Collectively, these results support the hypothesis that the LSO, not the MSO, produces the DN1 component of the BIC ABR.

In this study, we added results from rhesus macaques, which were chosen as a link between small-sized rodents and humans. Statistical analysis of the width of the DN1 amplitude versus ITD curves (Fig. 3*B*) indicates no significant differences between the species tested. This result provides evidence that the brainstem circuit that produces the BIC is conserved across these species despite their vastly different head sizes, and the nearly one order of magnitude differences in available ITDs. This further supports the notion that BIC DN1 is not linked to the range of naturally occurring ITDs for any given species.

Provided the monkeys were deeply anaesthetized, DN1 was identifiable in one measurement session but required 3000 signal repetitions per measurement condition. In the study of rodents by Benichoux et al. (2018), reliable DN1 was measured with 500 stimulus repetitions per condition, while human BIC studies have required between ∼8000 and 13,000 repetitions (Riedel and Kollmeier, 2006; Sammeth et al., 2020). Thus, while all species tested so far exhibit the same BIC DN1 characteristics, acquisition of reliable measures of DN1 across these species is quite different.

There are important differences between humans, macaques, and the smaller mammals that might provide an explanation for the apparent differences in the BIC’s measurability: (1) in animals with larger heads the neural generators of the BIC are farther from the electrodes and (2) in different species the volume of the MNTB (Medial nucleus of the trapezoid body)-LSO nuclei is slightly smaller relative to the total auditory brainstem volume (Glendenning and Masterton, 1998). For the most part, the relative size of the MNTB-LSO complex is fairly consistent except for the very smallest species (mice and bats) where the MNTB-LSO is relatively large, at least in the 53 mammalian species examined by Glendenning and Masterton (1998). This suggests that the size of the MNTB-LSO might not be able to explain the reduction in reliability of evoked DN1 in the Macaque and human relative to other species, and thus the size of the head may be more significant.

Further measurements that quantify the differences in BIC measurability will be necessary to further explore these issues. For example, it is possible that the total numbers of MNTB-LSO neurons in a given species is more important that the relative volume of these nuclei. However, detailed studies of numbers of neurons comprising all auditory brainstem nuclei in many mammalian species are lacking, and none conducted by the same set of researchers in the same laboratory, such as the Glendenning and Masterton (1998) database.

In conclusion, we examined the BIC in rhesus macaques, a primate with a head size intermediate between small rodents and humans. We found that the overall form of the function relating BIC DN1 amplitude to ITD appeared statistically indistinguishable from smaller rodents, suggesting that the brainstem circuitry that produces the BIC is largely conserved across these taxa. We also note apparent differences in the measurability of the BIC in different species. We suggest that head size is the most likely explanation for these differences, however further measurements that quantify measurability in different species will be necessary to prove this.

## Synthesis

Reviewing Editor: David Schoppik, New York University - Langone Medical Center Decisions are customarily a result of the Reviewing Editor and the peer reviewers coming together and discussing their recommendations until a consensus is reached. When revisions are invited, a fact-based synthesis statement explaining their decision and outlining what is needed to prepare a revision will be listed below. The following reviewer(s) agreed to reveal their identity: Jan Schnupp.

The reviews were quite positive, with only minor textual / graphical concerns that I’ll list below. Kindly make the requested changes/edits and we’ll move forward. Thanks for the submission & have a great weekend -- congrats on some nice work.

R1:

The manuscript providing data for the Rhesus macaque monkey adds to our knowledge on the size of the Binaural Interaction Component (BIC) and its relation to ITD. It confirms earlier results from Benichoux et al. (2018) and Laumen et al. 2016 indicating that the relation between BIC and ITD is similar across different species.

Some remarks on the results:

Figure 1 should show units for the y-axis also. Without that, the size of the ABR and BIC in the macaque monkey remains unclear. The same applies to Figure 2A-D (arbitrary units) and Figure 3B. Thus, the units must be added.

Some remarks on the discussion:

Since the BIC relates to the spiking activity in LSO, the discussion should also look at the relation between LSO size (not relative size) plus distance of the recording electrode from the LSO and size of the BIC.

The distance between the source of the BIC and the recording electrodes appears to be very important. Thus, the reader (and I) may ask what the attenuation of the potential may be in relation of the distance from the source (LSO).

Minor isues:

Please add information on the distance between the macaque ears (or maximum ITD).

How does the noise floor of the recording equipment affect the reliability in the comparative view? What is the minimum BIC that was measurable in the present manuscript.

R2:

Sig statement: “statistically identical” is an odd/awkward phrase, better avoided. There is nothing approximate or statistical about identity in mathematics. “Similar, and, based on data available so far, statistically indistinguishable” would be a far better way to dewscribe the data. The ANOVA on the data from Fig 3B is somewhat underpowered for the purpose of making a very rigrous cross-species comparisons. The results in this paper are perfectly fine, but given the huge variance and modest N of data from other studies shown in Figure 3B, the authors should perhaps refrain from making what seem like very strong pronouncements on whether the BIC is really “identical” across mammalian species. For the same reason, please reword “...statistically the same as...” in the Conclusion with something a little more cautious.

"temporary early-conductive”: should this not be “temporary, early, conductive”?

Conclusion: “Our data also provides further support to the hypothesis that the BIC DN1 is produced by the LSO and not the MSO.” I am sorry, but I do not see how the methodology would allow putative LSO and MSO contributions to be distinguished in a manner that is anything more than speculative. Sure, the data is like data from other species, and all these data can be interpreted in a manner that is not incompatible with the idea that the LSO may be a key generator. But reporting data which are “similarly not incompatible with” a hypothesis is not the same as “providing support for” that hypothesis. To provide real support, the study would have to have the potential of falsifying the contrary hypotheses that perhaps the LSO is not really where it’s all at. I don’t think it does. There are no conditions which would demonstrably disadvantage either the LSO or the MSO, for example, perhaps by deactivation. Nor can one conclusively discount possible contributions from stations downstream of the superior olive. This study was not designed to settle any questions about the origin of the interaction comppnent, so why are teh authors sticking their necks out and weighing in on that debate? Unless the authors can greatly strengthen the link between this particular conclusion and the data and analyses presented, I would strongly suggest deleting this sentence from the conclusion section. At the very least it will need to be reworded in a much more cautious fashion.

The font size of the figure and axis labels are on the small side. In fig 3 they are definitely too small.

## Author Response

The reviews were quite positive, with only minor textual / graphical concerns that I’ll list below. Kindly make the requested changes/edits and we’ll move forward. Thanks for the submission & have a great weekend -- congrats on some nice work.

We’d first like to thank the editor and reviewers for their positive comments, as well as for the kind comments above. We have attempted to address all concerns as indicated below.

R1:

The manuscript providing data for the Rhesus macaque monkey adds to our knowledge on the size of the Binaural Interaction Component (BIC) and its relation to ITD. It confirms earlier results from Benichoux et al. (2018) and Laumen et al. 2016 indicating that the relation between BIC and ITD is similar across different species.

Some remarks on the results:

Figure 1 should show units for the y-axis also. Without that, the size of the ABR and BIC in the macaque monkey remains unclear. The same applies to Figure 2A-D (arbitrary units) and Figure 3B. Thus, the units must be added.

This has been added.

Some remarks on the discussion:

Since the BIC relates to the spiking activity in LSO, the discussion should also look at the relation between LSO size (not relative size) plus distance of the recording electrode from the LSO and size of the BIC.

The distance between the source of the BIC and the recording electrodes appears to be very important. Thus, the reader (and I) may ask what the attenuation of the potential may be in relation of the distance from the source (LSO).

We agree that total neuron number in a nucleus is probably the more important variable. Unfortunately, these data do not exist in large numbers of mammals. For example, we are not aware of studies that have measured neuron numbers in all relevant brainstem nuclei at the same time in the same lab. Thus, the best database continues to be the Glendenning and Masterton (1989) study where the same people in the same lab examined 10 brainstem nuclei in 53 mammalian species. The only data they supply is nucleus volume relative to total auditory brainstem volume. In terms of the MNTB-LSO nuclei, the human is not deviant from most other mammals. However, the mouse is deviant in the sense that the MNTB-LSO is exceptionally larger than other mammals and the MSO is basically unidentifiable.

Second, the distance of these nuclei to the locations of the recording electrodes is not known. Presently, head diameter is the best proxy for at least a relative distance across the species - the electrodes in mouse are most certainly closer to the generator than the electrodes in human.

Minor isues:

Please add information on the distance between the macaque ears (or maximum ITD).

The head width was already mentioned in the methods section. All of the macaques we used had an interaural diameter of around 67 mm. The widest head measurements we have was 68 mm. They didn’t vary too much, and the mean is 67. We made this clearer in the methods section.

"The average interaural diameter did not vary much across individuals, with the mean being ∼67 mm.”

How does the noise floor of the recording equipment affect the reliability in the comparative view? What is the minimum BIC that was measurable in the present manuscript.

The same equipment and electrodes were used in all of our experiments, from mouse to human. In our case, we found it necessary to use 500 averages in small rodents, 3000 in monkey and 8000-12000 in human. It seems to take more signal averaging to recover the BIC in the larger mammals. Because the equipment and methodology was the same, it seems likely that the recording equipment were not the limiting factor.

R2:

Sig statement: “statistically identical” is an odd/awkward phrase, better avoided. There is nothing approximate or statistical about identity in mathematics. “Similar, and, based on data available so far, statistically indistinguishable” would be a far better way to dewscribe the data.

We have modified this statement using your suggested text.

The ANOVA on the data from Fig 3B is somewhat underpowered for the purpose of making a very rigorous cross-species comparisons. The results in this paper are perfectly fine, but given the huge variance and modest N of data from other studies shown in Figure 3B, the authors should perhaps refrain from making what seem like very strong pronouncements on whether the BIC is really “identical” across mammalian species. For the same reason, please reword “...statistically the same as...” in the Conclusion with something a little more cautious.

We have changed this throughout to be more cautious. We tried to be cautious already about saying it is conserved across mammals, since we only really have rodents and primates. We have read through the manuscript and tried to change these statements to read more cautiously.

We have reused your previous suggestion for wording, and tried to phrase it as the bic vs ITD curve is statistically indistinguishable across species measured, and that the brainstem circuitry producing the BIC appears likely to be conserved across a wide range of mammalian taxa.

"temporary early-conductive”: should this not be “temporary, early, conductive”?

This is indeed awkward phrasing. We have changed this tto:

”...and autism spectrum disorders (ElMoazen et al., 2020)⁠, as well as in individuals with early conductive (Gunnarson and Finitzo, 1991)⁠ and normal age-related hearing loss (Van Yper et al., 2016)⁠.”

Conclusion: “Our data also provides further support to the hypothesis that the BIC DN1 is produced by the LSO and not the MSO.” I am sorry, but I do not see how the methodology would allow putative LSO and MSO contributions to be distinguished in a manner that is anything more than speculative. Sure, the data is like data from other species, and all these data can be interpreted in a manner that is not incompatible with the idea that the LSO may be a key generator. But reporting data which are “similarly not incompatible with” a hypothesis is not the same as “providing support for” that hypothesis. To provide real support, the study would have to have the potential of falsifying the contrary hypotheses that perhaps the LSO is not really where it’s all at. I don’t think it does. There are no conditions which would demonstrably disadvantage either the LSO or the MSO, for example, perhaps by deactivation. Nor can one conclusively discount possible contributions from stations downstream of the superior olive. This study was not designed to settle any questions about the origin of the interaction comppnent, so why are teh authors sticking their necks out and weighing in on that debate? Unless the authors can greatly strengthen the link between this particular conclusion and the data and analyses presented, I would strongly suggest deleting this sentence from the conclusion section. At the very least it will need to be reworded in a much more cautious fashion.

We did have part of the discussion dedicated to this as it was a major part of the Benichoux et al. Study, and so included a mention of that in the conclusion. But you are quite right that this study is not designed to settle that question. We decided it was easiest just to remove this particular sentence.

The font size of the figure and axis labels are on the small side. In fig 3 they are definitely too small.

We have increased the text size in all the figures and reworked to make as readable as possible. All figures are in a scalable format, and we will work with the journal to ensure the published version is as readable as possible
